# Brain Imaging and Phenotyping for the China Phenobank Project

**DOI:** 10.1007/s43657-024-00197-3

**Published:** 2025-01-02

**Authors:** Wenjia Bai

**Affiliations:** 1 Department of Computing, Imperial College London, London, SW7 2AZ UK; 2 Department of Brain Sciences, Imperial College London, London, W12 0NN UK

Large populations with multi-modal biomedical phenotypes are crucial for investigating the potential causes of human diseases. In particular, large population-based imaging studies can provide detailed imaging-derived phenotypes (IDPs) to quantitatively describe the anatomical structure and function for assessing their associations with genetics, life-style factors and disease outcomes. However, it is not a trivial task to initiate a large population-based imaging study, considering the challenges of logistics, cost, standardisation of the image acquisition protocol, robust image analysis pipelines for IDP extraction and quality control etc. Published in Phenomics, Wang et al. describe the brain image acquisition and phenotyping protocol for the China Phenobank Project (CHPP) (Wang et al. 2023), an effort to assess and understand the current health status of a Chinese population, as well as to investigate the causes and progression of common diseases in China.

By far, more than 1000 participants have been scanned at their first imaging visit, as a pilot phase for CHPP (Phase I). This pilot cohort size may be relatively smaller compared to the UK Biobank Project (UKB) (Littlejohns et al. 2020) or the German National Cohort (GNC) (German National Cohort (GNC) Consortium 2014), that has already collected tens of thousands imaging scans. However, considering CHPP was initiated in 2017, later than either UKB or GNC, as well as the population size of China, the CHPP cohort size could potentially be scaled up in the next five to ten years following the pilot phase.

In terms of image acquisition protocols, CHPP collects the cardiac, brain and abdominal magnetic resonance (MR) images similar to UKB, as well as the uterus, prostate MR and lung computed tomography (CT) that are not covered by UKB. When we look at the brain MR imaging protocol, CHPP uses 3.0-Tesla (3T) Philips scanners with a 64-channel head coil (Philips Ingenia, Amsterdam, Netherlands), whereas UKB runs three imaging centres in parallel that are all equipped with 3T Siemens scanners with a 32-channel receive head coil (Siemens Skyra, software VD13). The image acquisition durations are similar. The CHPP brain imaging protocol takes 30 min (Wang et al. 2023), whereas UKB brain imaging takes 31 min (Alfaro-Almagro et al. 2018). They both cover T1, T2 FLAIR, diffusion MRI (dMRI), and resting-state functional MRI (rfMRI). The differences are that CHPP also includes magnetic resonance angiography (MRA), but does not include susceptibility-weighted MRI (swMRI), task functional MRI (tfMRI), or arterial spin labelling MRI (ASL) (Alfaro-Almagro et al. 2018). The coverage of T1, T2 FLAIR, and diffusion MRI (dMRI) allows CHPP to derive structural and connectivity IDPs, such as the grey and white matter volumes, white matter hyperintensities (WMH) volume, cortical thickness, the fractional anisotropy (FA) etc., using FSL and FreeSurfer tools in a way similar to how IDPs are derived from the UKB cohort. The additional MRA sequence facilitates the characterisation of the cerebral vascular structures.

A repository of brain IDPs derived from CHPP will serve as a valuable and unique resource for assessing the brain structure and function with a wide range of non-imaging factors and disease outcomes in the Chinese population. It will also facilitate cross-validation of clinical findings in different ethnic groups, when being used together with other resources such as UKB or GNC that focus more on the European population. One notable point is that the CHPP population at the pilot phase are between 20 and 60 years old that spans across young, mid and late ages, whereas the UKB population were aged between 40 and 69 years old i.e. in their mid- to late ages when being recruited (Allen et al. 2024). This may allow CHPP to monitor the longitudinal changes of brain IDPs and health outcomes across a longer life span in the follow-ups compared to UKB. At the meantime, this also means it might take longer time for CHPP to accrue the number of cases for certain diseases that are more common in old populations.

While the authors' work confirms the feasibility of large-scale brain imaging and phenotyping for CHPP, there are also some practical tasks still ongoing to further support the usability of this data resource. For example, the data management system and the multi-omics database are currently under development. The linkage of imaging data to health outcomes of the participants might take some time. A population-level database that integrates both imaging and multi-modal non-imaging data would be highly valuable for pushing innovative scientific research, such as for discovering phenome-wide associations of brain imaging phenotypes (Miller et al. 2016; Wang et al. 2022), for investigating causal relationships between the brain and the heart using Mendelian randomisations (Francis et al. 2022; Zhao et al. 2023), or for identifying relationships across multiple organs and diseases (Tian et al. 2023; Nauffal et al. 2024). The practical challenges in multi-modal database construction are addressable by a coordinated working group with multi-domain expertise. Here the work in (Wang et al. 2023) offers an exciting first glimpse of the incoming large-scale phenobank for the Chinese population and describes the richness of its IDPs for conducting health-related research that can benefit the entire research community.

## Data Availability

Not applicable.
